# Advances in bio-active constituents, pharmacology and clinical applications of rhubarb

**DOI:** 10.1186/s13020-017-0158-5

**Published:** 2017-12-28

**Authors:** Yu-Jie Cao, Zong-Jin Pu, Yu-Ping Tang, Juan Shen, Yan-Yan Chen, An Kang, Gui-Sheng Zhou, Jin-Ao Duan

**Affiliations:** 1 Jiangsu Collaborative Innovation Center of Chinese Medicinal Resources Industrialization, and Jiangsu Key Laboratory for High Technology Research of TCM Formulae, and National and Local Collaborative Engineering Center of Chinese Medicinal Resources Industrialization and Formulae Innovative Medicine, Nanjing University of Chinese Medicine, Nanjing, 210023 Jiangsu China; 2 College of Pharmacy and Shaanxi Collaborative Innovation Center of Chinese Medicinal Resources Industrialization, Shaanxi University of Chinese Medicine, Xianyang, 712046 China

**Keywords:** Rhubarb, Ingredients, Pharmacological activities, Clinical application, Functional mechanism

## Abstract

Rhubarb is one of the most ancient, commonly used and important herbs in Chinese medicine. The modern researches of rhubarb clarified the efficacies, ingredients and mechanisms in a more scientific and rigorous way. The main chemical compositions of rhubarb include anthraquinones, anthrones, stilbenes, tannins, polysaccharides etc. These compositions show extensive pharmacological activities including regulating gastrointestinal, anticancer, antimicrobial, hepatoprotective, anti-inflammatory, protecting cardiovascular, cerebrovascular and so on. This paper reviews the recent studies on the active ingredients, pharmacological effects, clinical application and functional mechanism.

## Background

Rhubarb is a collective name of various perennial plants of the genus *Rheum* L. from Polygonaceae family. This plant has important economic value, not only referred to a few edible rhubarbs [1], but also used as purgative drug in China since the third millennium BC [2], firstly recorded in *Shen Nong’s Herbal Classic*. Rhubarb has been suggested to exert eliminating heat, purging fire, cooling blood, dispersing blood stasis, dredging collateral antidotal and purgative effects, used to treat constipation, diabetic nephropathy, chronic renal failure, acute pancreatitis, gastrointestinal bleeding and other diseases [3].

There are articles summarizing the research progresses of rhubarb on treating acute organophosphorus pesticide poisoning [4], acute ischemic stroke [5], acute pancreatitis [6], chronic kidney disease [7] in recent years. Zheng [8] summarized the researches of rhubarb containing the isolation, pharmacological activities, and phytochemical analysis. But there is no article to associate the different components of rhubarb with diseases. In this article, we not only introduce the active ingredients, pharmacology, applications and mechanism of rhubarb, but also summarize the relationship between ingredients and pharmacologic action.

## Chemical components

Back in the early years of the nineteenth century, the chemical compositions of officinal rhubarb had been researched [3]. In recent years, unofficial rhubarbs with rich resources are also studied. Although they are different species, the main composition of these rhubarb species is similar. Scientists and medics isolated various types of compounds from rhubarb, containing anthraquinones and their glycosides, anthrones and their glycosides, stilbenes, butyrophenones and chromones, tannins, saccharides and so on [9].

### Anthraquinones

**Fig. 1:**
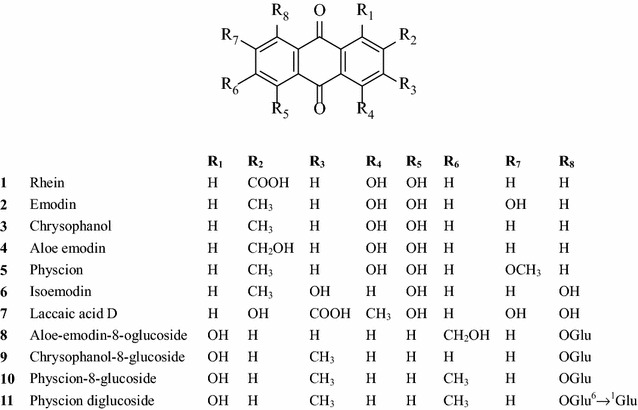
Main structures of rhubarb anthraquinones

Anthraquinones are the main characteristic as well as pharmacodynamic ingredients of rhubarb [10, 11]. The proportion of anthraquinones ranges from 3 to 5% in different species [3]. More than 30 anthraquinones have been isolated and identified from rhubarb [12]. They are divided into free type and combination type. Free anthraquinones mainly contain rhein, emodin, aloe-emodin, chrysophanol, physcion, isoemodin, chrysaron, isoemodin, laccaic acid D. Combination anthraquinones are the glycosides combined by free anthraquinones and glycosyl. There are many kinds of anthraquinone glycosides, containing aloe-emodin-8-glucoside, emodin-8-glucoside, rhein-8-glucoside, physcion diglucoside, emodin-6-glucoside etc. [3, 9]. Main structures of anthraquinones (1–11) are as follows (Fig. 1) [3, 8, 12].

Rhein has the ability of protecting kidney [13], inhibiting the formation of renal fibrosis [14], improving diabetic nephropathy [15] and lipid disorders [16]. Rhein also has a strong inhibitory effect on common clinical anaerobes [17].

Emodin has a wide range of pharmacological effects, including anti-tumor [18], anti-microbial [19], antioxidant [20], anti-inflammatory [21]. It also can bring high blood pressure down, decrease blood lipids and improve microcirculation, protect liver and kidney [17].

Chrysophanol has a protective effect on the nervous system, improving the activity of antioxidant enzyme, reducing the damage of oxygen free radicals to cells [22].

Physcion also has neuroprotective effect, inhibiting the inflammatory response after cerebral ischemia and reducing the nerve damage caused by reperfusion [23]. Besides, physcion has anti-tumor effects on a variety of carcinoma cells, mainly through inhibiting cell proliferation, inducting apoptosis and blocking cell cycle [24].

Aloe-emodin, another important active compound of rhubarb, has attracted much attention, due to its various effects such as cardiovascular protection, hepatoprotective activities, anti-tumor, antibacterial, antifungal, antiviral, anti-inflammatory, immune regulation, laxative [17, 25].

Anthraquinone glycosides process the characteristic of antioxidant, anticancer, anti-inflammatory, laxative and many others biological properties [26], laxative activity strongest among them.

### Anthrones and dianthrone

**Fig. 2:**
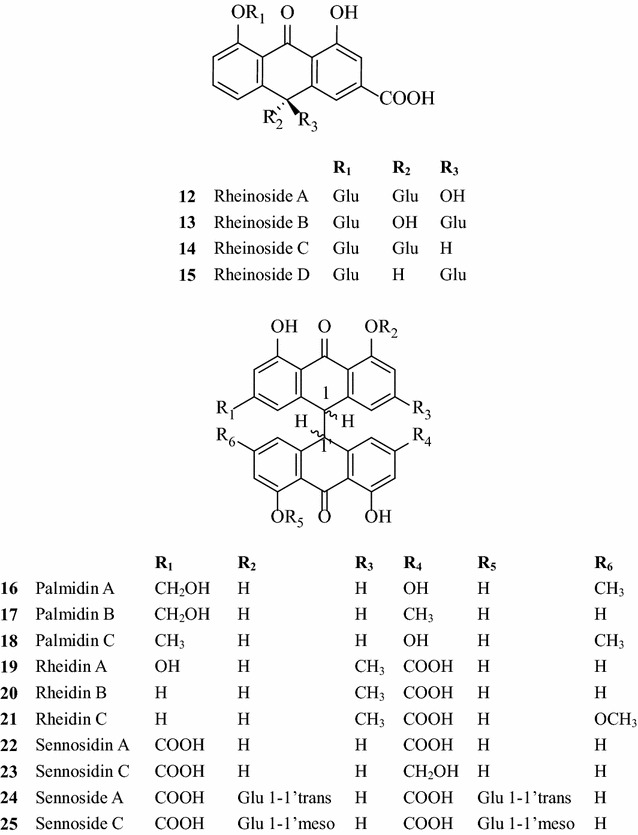
Main structures of rhubarb anthrones and dianthrones

Anthrones and dianthrone, also characteristic components of rhubarb, are related to purgative activity. Mainly these include rheinosides A–D, palmidin A, B, C, rheidin A, B, C, and sennosidin A–F, etc. [9]. 26 anthrones have been isolated from the species of this genus [8]. Sennosides have a strong cathartic effects though translating to anthraquinones in vivo. The main structures of anthrones and dianthrone (12–25) are as follows (Fig. 2) [3, 8, 12].

### Stilbenes

**Fig. 3:**
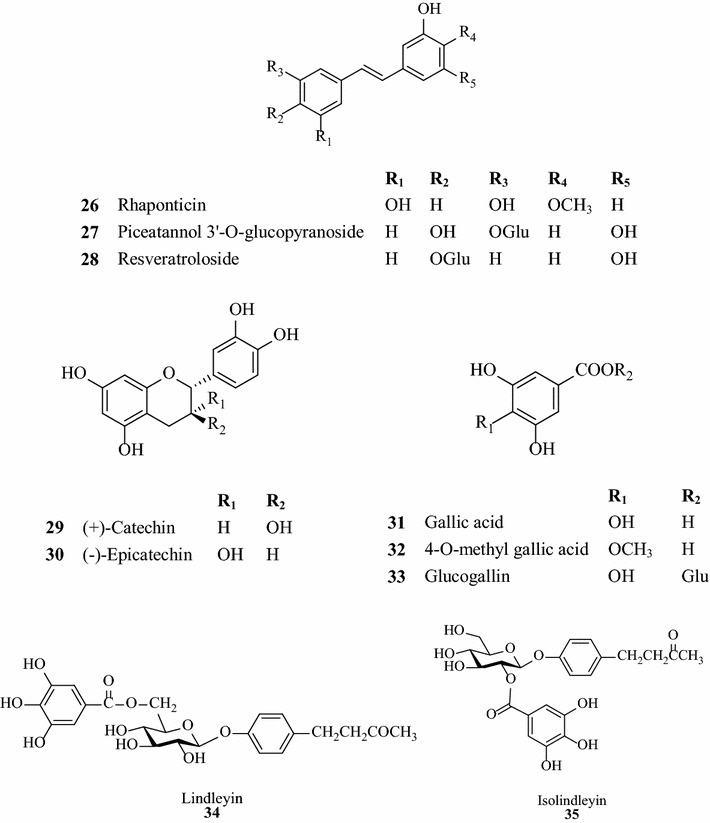
Main structures of rhubarb stilbenes, tannins, butyrophenones and chromones

Stilbenes are important components of rhubarb, concerning antihyperlipidemic, antioxidant and hepatoprotective effect [3, 27]. So far, there are 31 compounds found in rhubarb belonging to stilbenes [12], such as rhapontigenin, isorhapontigenin and rhaponticin. Some representative structures of stilbenes (26–28) are showed at Fig. 3 [3, 8, 12].

### Tannins

Since the 1880s, the discovery that rhubarb tannins reduced the content of BUN, sparked great interest and attention of scholars both at home and abroad. Tannins in rhubarb generally account for 10–30% [28]. It can be divided into hydrolytic type and condensation type. Gallic acid and d-cate-chin are the monomers of these tannins. Studies have discovered that tannins are the active elements owing to the stypticity and constipate activity of rhubarb [29]. It has been proved that tannins can adjust genotoxicity, oxidative stress, inflammation and apoptosis [30]. Total tannins extract can protect the kidney of K_2_Cr_2_O_7_-injured rats by treating CrNT as a free radical scavenger [31]. The basic structures of tannins (29–33) recorded at Fig. 3.

### Butyrophenones and chromones

6 butyrophenones and 14 chromones have been isolated from rhubarb already. Lindleyin and Isolindleyin whose structures are showed at Fig. 3, have been confirmed possessing anti-inflammatory and analgesic activity [32]. Chromones are of expanding coronary vessels, decreasing blood pressure, removing cholesterol, antibacterial and other activities [3].

### Polysaccharides

Polysaccharides play multiple roles and have extensive bioactivities in life process, with an immense potential in healthcare, food and cosmetic industries, due to their therapeutic effects and relatively low toxicity [33]. It has been proved that rhubarb polysaccharides have the following pharmacological activity, lowering the blood sugar, protecting liver, promoting the proliferation of intestinal epithelial cell, antineoplastic, anti-senescence and etc. [34].

**Fig. 4:**
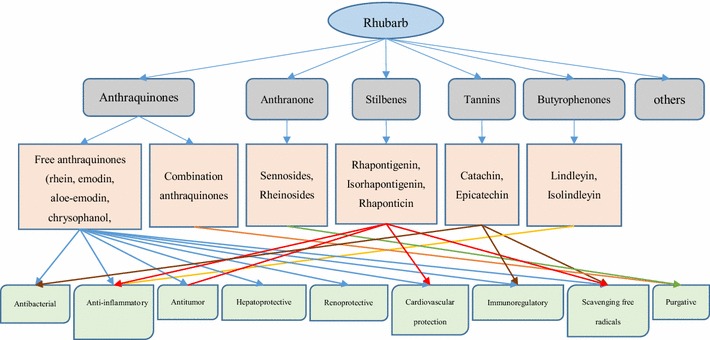
Relationship between ingredients and efficacy of rhubarb

We list the main active ingredient groups, representative component and mainly pharmacological activity, showing the relationship between these components and effects. Rhubarb contains several different active ingredients. Each active ingredient often has different pharmacological activities, acting on multiple targets. One pharmacological activity may also be caused by a variety of ingredients. There is a synergistic effect among these components. The relationship is summarized in Fig. 4.

## Pharmacology

### Digestive system

#### Purgative

Rhubarb has been used as a first-choice herb for constipation in clinic for thousands of years in China. Its purgative activity is definite. Processing can change the potency and efficacy of Chinese herb. The purgative activity is different in raw rhubarb and its processed products [35].

**Fig. 5:**
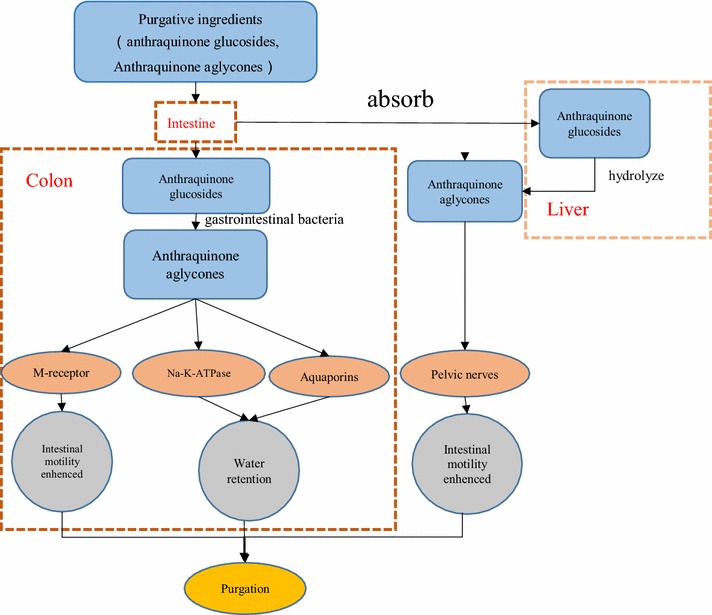
Active constituents and mechanism of rhubarb exerting purgative activity

Active ingredients and mechanism of purgation: Combination anthraquinones, including sennosides, rheinosides and anthraquinone aglyconesa, are considered as the main bioactive constituents of the laxative effect [26, 36], playing the most important role in stimulating the intestine and leading to diarrhea [37]. Combined anthraquinone is metabolized into free metabolise in intestinal canal to exerting laxative effect [38]. Sennoside A, the strongest purgative composition, is rarely absorbed in the intestine, most of them reached the colon, metabolized into rhein anthrone and rhein in the intestine [39]. After giving chloramphenicol, the active of *Escherichia coli* restrained, the purging effect of sennoside A and C weaken, anthrone in the colon is also greatly reduced [40]. When the free anthraquinone derivatives were injected to the colon of rat, the re-absorption of water and electrolyte would be inhibited, resulted in diarrhea [41]. Most of free anthraquinones are absorbed before arriving colon. Therefore, combined anthraquinones play drastic effect by means of metabolizing into free anthraquinones. It is thought that anthraquinones can stimulate the nerve plexus within the mucosa and intestinal smooth muscles, promoting peristalsis [9]. The rhubarb extractives and the anthraquinone derivatives can antagonize the adrenaline effectively, which can inhibit the contraction of the smooth muscle in vitro system of isolated intestine [37]. It also regulates the colon cholinergic neuron of constipating rats [42]. Besides, Rhubarb effectively down-regulates the expression of AQ4P in rat’s proximal colon, and rhein/emodin can suppress the AQ4P expression of LoVo cells in vitro [43]. Sennoside A may decrease AQP3 expression in the colon to inhibit water transport from the luminal to the vascular side, leading to laxation [39]. The mechanism of its purgative activity is summarized as Fig. 5 [3, 9, 38, 44, 45].

#### Hepatoprotective

Liver-protective function is an important part of studies on rhubarb in recent years. Rhubarb anthraquinones and tannins have a biphasic effect on liver, protection and damage. Anthraquinones showed stronger improvement on liver fibrosis and liver cell injury than tannins, and high dose tannins may injury liver in some extent [46]. Rhubarb and its free anthraquinones are also investigated in hepatic encephalopathy [47], liver fibrosis [48, 49], intrahepatic cholestasis [50] and so on.

### Cardiovascular system

#### Promoting blood circulation and hemostasis

Rhubarb not only processes the effect of hemostasis but also improves hemorheology, stopping bleeding without leaving congestion. Charred rhubarb significantly improved the plasma viscosity, hematocrit, FIB, PT, APTT and TT in the acute blood stasis rats [51], owning the best effect.

Mechanism of promoting blood circulation and Hemostasis: It is confirmed that chrysophanol and tannin are associated with the hemostasis effect. Chrysophanol can short the time of clotting, increase the number of platelets, promote blood coagulation in mice and tannin has a convergence effect, to promote local vasoconstriction [52]. Zhang found the main hemostasis mechanism of micron rhubarb charcoal was to produce prothrombin and thrombin by activating both endogenous and exogenous blood coagulation factor [53]. It is also believed that the pharmacological basis of promoting blood circulation is inhibiting the activity of Na^+^-K^+^-ATPase, to increase the plasma osmotic pressure, leading the water in the organization transferring into the blood vessels, increasing blood volume, helping with relieving microcirculation disturbance [54].

#### Hypolipidemic

Rhubarb has the effect of preventing and treating high blood lipids, reducing the TC, LDL, TG levels caused by high fat diet [55]. Rhubarb polysaccharide can reduce the blood lipid of diabetic atherosclerosis rats [56]. It has been confirmed that rhein and rheum emodin can regulate blood lipids, inhibit the formation of hyperlipidemia, prevent atherosclerosis [56, 57].

Mechanism of hypolipidemic: It promotes the excretion of cholesterol, improves blood rheology characteristics, decreases the release of inflammatory factors to maintain the balance of fat metabolism, inhibits the synthesis of cholesterol and triglyceride to achieve the purpose of regulating blood lipids [58]. Chen [59] found chrysophanol can reduce the blood lipid levels of zebrafish after high-fat diet, the mechanism may reduce the absorption of lipids in intestinal tract.

### Urinary system

#### Kidney protection

Kidney protecting effect of rhubarb is owing to the combination of various pharmacological activities itself. Rhubarb, with anti-bacterial, anti-inflammatory, enhancing immunization, diuretic, regulating metabolism and other effects, protects the damaged kidney tissue, promotes protein synthesis, speeds up the excretion of waste and so on [3]. Rhubarb is commonly used in clinical practice to treat kidney disease such as chronic functional failure and diabetic nephropathy.

Mechanism of kidney protection: In recent years, the renal protective effect of rhubarb [60] and its active components, especially emodin [61] and rhein [62, 63], have raised widespread concern. But generally accepted views on the therapeutical mechanism have not been attained [64]. Rhein has the effect of improving the metabolism of glycolipid, reducing the excretion of urinary protein and anti-oxidative stress, which may be one of the mechanisms to prevent and treat diabetic nephropathy [65]. Emodin mainly involves cells and cytokines, such as TGF-β, CTGF, MCP-1, and TSP-1 to achieve the purpose of treating kidney diseases [66].

#### Nervous system

Rhubarb exerts an antipyretic effect by acting on the central nervous system. The mechanism of rhubarb antipyretic effect may be related to deduct the production of PGE_2_ and cAMP in hypothalamus and down-regulate central body temperature [67]. Chrysophanol improved the learning and memory function of cerebral ischemia–reperfusion injured mice, increasing its anoxia tolerance capability, repairing the damage of brain tissue pathomorphism, so as to protect brain [68]. Besides the central nervous system, rhubarb also acts on the peripheral nervous system. It can improve the cholinergic nerve decreasing caused by constipation in colonic myenteric plexus [42]. Chrysophanol and physcion significantly improved the activity of hypoxia-injured cells, enhanced cell survival rate, protected and improved the ultrastructure of hypoxia-induced neurons [69].

#### Respiratory system

Rhubarb is also used to treat acute respiratory distress syndrome in clinic. Rhubarb, combining with Shenfu injection, can improve the condition of patients with acute respiratory distress syndrome, such as improving organ dysfunction and respiratory mechanics index, increasing the oxygenation index, shortening mechanical ventilation time [70]. Rhubarb also has a good therapeutic effect on respiratory failure caused by other diseases.

### Others

#### Anti-inflammatory

Rhubarb has the effect of heat-clearing and detoxifying for its anti-inflammatory effects, used to treat inflammations caused by a variety of reasons. Rhubarb remitted the degree of auricular swelling and foot swelling in mice and rats, reduced total protein and LTB4 in airbag synovitis exudates [71].

Mechanism of anti-inflammatory: Currently, studies of rhubarb anti-inflammatory mechanism are focused on its monomer components such as emodin, rhein and aloe-emodin. Eodin can reduce the level of IL-6 in periodontal tissue of periodontitis rats [72]. Due to intervention effect of emodin lipid nano-microbubble, the protein expressions of p-P38, p-ERK, p-JNK and the release levels of inflammation cytokine, such as TNF-α, IL-1β, IL-6, were significantly decreased [73]. Rhein reduced the level of inflammatory factors caused by type II diabetes mellitus [74]. Hu [75] found aloe-emodin was also the bioactive component of rhubarb related to anti-inflammatory effect. Aloe-emodin could decrease the production of proinflammatory cytokine in LPS-induced RAW264.7 macrophages by inhibiting NF-κB, MAPK, and PI3K pathways.

#### Antitumor

A number of studies have shown that the anthraquinones of rhubarb could inhibit the growth and proliferation of various cancer cells [8]. Its extract and active monomers including aloe-emodin, emodin and rhein, are researched on multiple cancer models [76–78].

Mechanism of antitumor: to determine and identify the possible molecular mechanisms of anti-cancer effect of rhubarb, medical practitioners focused on the free anthraquinones of rhubarb and emodin is the most frequently studied element among the active constituents. Firstly, emodin inhibited the proliferation of tumor cell and induced apoptosis. Liu [79] found emodin inhibit the growth of human pancreatic cancer cell line Panc-1, and this inhibition effect was obvious connected with concentration and time. Secondly, it could inhibit the growth of tumor blood vessels. He [80] investigated the 95% ethanol extract and four subsequent fractions of rhubarb root and five anthraquinones extract on zebrafish model by quantitative endogenous alkaline phosphatase assay and staining assay, finding the anthraquinones with acidic or polar, hydrophilic substitution at C-6 or C-3 positions, played a substantial role in inhibiting angiogenesis. Then, rhubarb inhibited carcinoma cell metastasis. Tsai found rhubarb extract inhibit HA22T cell migration in wound healing in a dose-dependent manner [81].

#### Anti-bacterial

It has been confirmed that anthraquinone derivatives of rhubarb have remarkable antibacterial activity on several experimental bacterial strains in vitro [82, 83]. *Staphylococcus aureus* are often used on experiment because it is sensitive to rhubarb. Zhou [84] proposed rhubarb exerted antibacterial activity by changing the membrane permeability, inhibiting the synthesis of proteins and respiratory metabolism. Liu investigated on Emodin’s effect and mechanisms on anti-MRSA in vitro and vivo, discovering that Emodin can destroy the cytoderm and cytomembrane structure of methicillin-resistant *Staphylococcus aureus* [85].

#### Anti-fibrosis

Many studies showed that rhubarb and its active components principally refer to emodin and rhein, could ameliorate organ fibrosis including renal, liver, lung, pancreas, cardiovascular system and so on [86, 87].

Mechanism of anti-fibrosis: Xu [88] summarized the mechanism for rhein ameliorating renal fibrosis as follows: inhibiting the infiltration of inflammatory cell, the transdifferentiating of renal tubular epithelial cells, the expression of profibrotic cytokines and blocking the activation of interstitial fibroblast. Wang [49] investigated the inhibitory effect and possible mechanism of emodin on hepatic fibrosis caused by CCl_4_, finding that emodin exerted anti-hepatic fibrosis effect for inhibiting the activation of hepatic stellate cell though up-regulation the expression of Smad7 and down-regulation the expression of α-SMA in liver tissue. Liu [87] observed the influence of emodin on bleomycin-induced pulmonary fibrosis in rats, explored its protective mechanisms, proposing emodin may protect against rats with pulmonary fibrosis by enhancing antioxidation and anti-inflammatory ability.

## Application

With the deep research on rhubarb, a traditional diarrhea drugs, its clinical application is widened, not only used to treat the constipation, but also other diseases.

### Constipation

Rhubarb has a significant diarrhea effect, used to treat constipation caused by various reasons on clinic [89–91]. Yu found that umbilicus compressing of Rhubarb and Mirabilite achieved satisfactory and safe efficacy on constipation patients with orthopedic surgery by observing their bristol stool scale, defecation frequency and total effective rate [92]. Clinically, rhubarb often plays a curative effect through acupoint application rather than oral administration.

### Diabetic nephropathy and chronic renal failure

Rhubarb is one of the most popular traditional Chinese herbs used in treating diabetic nephropathy (DN) and chronic renal failure (CRF) [93].

A large number of related literatures reported that rhubarb was often used in conjunction with other drugs in the treatment of diabetic nephropathy. Liu [94] found the SCr, BUN, Alb and FBG significantly improved after the patients treated with rhubarb compound. Rhubarb could improve the condition of diabetic nephropathy patients by reducing the excretion of urinary protein, lowering blood lipid, improving renal function, regulating the abnormal expression of TGF-β1, MMP-2, MMP-9 and MCP-1 in the blood of diabetic nephropathy patients, inhibiting renal inflammation and fibrosis process [95].

Rhubarb soda tablets combined with Jinshuibao Capsule significantly reduced Scr and BUN levels in patients with chronic renal failure [96]. Xiao [97] summarized the clinical and laboratory study on the rhubarb in the treatment of CRF. He found rhubarb and its prescriptions had a definite curative effect on CRF by ameliorating azotemia, preventing nephritic compensatory hypertrophy and high metabolism situation, and so on.

### Acute pancreatitis

Rhubarb can promote the secretion and discharge of pancreatic juice, increase pancreatic juice flow. It is used to treat acute edema and hemorrhagic necrotizing pancreatitis [6].

Rhubarb and mirabilite external application reduced the risk of abdominal distension and stomachache, reducing the probability of complications in patients with acute pancreatitis. The relevant laboratory indicators were closer to normal and the average hospital stay was significantly shorter [98].

Rhubarb inhibited the inflammatory response, improved intestinal microcirculation and restored normal intestinal absorption, to exert the function of treating AP [99]. The free anthraquinone of rhubarb and the rhubarb decoction could attenuate kidney injury induced by acute pancreatitis [100].

### Gastrointestinal bleeding

Rhubarb has hemostatic effect, used to treat gastrointestinal bleeding clinically. Conventional treatment combined with rhubarb powder exerting better therapeutic effect on the upper gastrointestinal bleeding by comparing control group and observation group [101]. Tan found rhubarb reduced curative time and increased the recovery rate of gastrointestinal bleeding caused by severe brain injury [102].

### Others

In addition to the above diseases, rhubarb is also used to treat a variety of liver and kidney diseases, gastrointestinal dysfunction, cancers, hemorrhoid, periodontitis and so on [3].

### Toxicity

It has been confirmed that rhubarb has varying degrees of toxicity on liver, kidney, gastrointestinal tract, reproductive system and blood systems, may possess teratogenicity and reproductive toxicity [103]. It is thought that gastrointestinal tract, liver, kidney are potential target organs of its toxicity.

Gastrointestinal toxicity of rhubarb characterized by diarrhea, constipation and melanosis [104]. Anthraquinones in rhubarb have purgative effect, while tannic acid and other components causing diarrhea. Small doses of rhubarb did not cause diarrhea, on the contrary inducing secondary constipation after discontinuation [26].

Yan [105] administrated total rhubarb anthraquinones on S.D. rats for 13 weeks to induce nephrotoxicity, finding the renal tubule epithelial cells swelled and denatured in tissue slice. Da [106] compared the toxicity of rhein and emodin in human renal tubular epithelial cells (HK-2), discovering that both emodin and rhein induced the apoptosis of HK-2 cells, having significant cytotoxic effect and the cytotoxicity of emodin on HK-2 cells was stronger than Rhein.

Animal experiments and clinical application confirmed rhubarb has hepatoprotective and choleretic effect. However, under certain conditions, rhubarb may damage liver [47]. Wang [107] investigated the effect of total extracts from prepared rhubarb on normal and pathological animals within a high dose range, finding prepared rhubarb showed bidirectional effects in hepatoprotection and hepatotoxicity, which could protect liver in CCl_4_ injured chronic hepatic injury, but had a certain hepatotoxic effect to normal animals.

Besides, rhubarb is of reproduction toxicity. Wang [108] explored the abortion effect and mechanism induced by rhubarb extract. The result showed that rhubarb extract not only interfered the stability of pregnancy state in pregnant mice for its purgative activity, but also directly affected endometrial environment in early embryonic mice, resulting in abortion. Administrating rhubarb for 30 days induced a significant toxic effect on testis in adult rats by promoting interstitial cell apoptosis, affecting the synthesis of testosterone, reducing spermic formation, and the injury degree was dose-dependent [109].

## Conclusion and perspectives

Rhubarb is one of the oldest and most frequently used herbal medicines in China, Korea, Japan, and other Asian countries. In this article, we summarize the active ingredients, pharmacological effects, functional mechanisms of rhubarb, as well as its clinical applications. There are about 200 compounds isolated from rhubarb, including anthraquinones, anthrones, stilbenes, flavonoids, acylglucosides, and pyrones. Most of the studies focused on exploring the bioactivities of anthraquinones. These constituents have shown extensive pharmacological activities, including cathartic, anticancer, hepatoprotective, anti-inflammatory, anti-microbial, analgesic effects and so on.

Pharmacological effects of rhubarb are extensive. In clinic, it is used to treat various diseases, such as constipation, acute pancreatitis, gastrointestinal bleeding, DN, CRF and so on. But rhubarb is not suitable for long-term using to avoid producing toxic and side effects. It is an important direction for the development of rhubarb that reducing its toxicity, through compatibility, processing, changing the dosage or administration route et al., to play a better therapeutic role.

Main active ingredients of rhubarb, rhein, emodin, chrysophanol and so on, have extensive pharmacological activities with low toxic and side effects, possessing good application prospects. At present, rhein is studied as a drug candidate to treat cancer. The potential anti-tumor mechanisms may block cell cycle, induce apoptosis and control metastasis. Some scholars are also committed to develop rhein as a new drug for diabetic nephropathy. Lindleyin whose pharmacological action is similar to aspirin, is expected to be developed as a new anti-inflammatory and analgesic drug. Developing and applying monomer compounds divided from rhubarb is another development direction of rhubarb.

## Abbreviations

TCM: Traditional Chinese Medicine
ALT: alanine aminotransferase
AST: aspartate transaminase
LPS: lipopolysaccharide
MAO: monoamine oxidase
GSH-ST: glutathione *S*-transferase
α-SMA: alpha smooth muscle actin
P-gp: *P*-glycoprotein
AP: acute pancreatitis
TNF-α: tumor necrosis factor-α
IL-1β: interleukin 1β
IL-6: interleukin 6
TC: total cholesterol
LDL: low density lipoprotein
TG: triglyceride
CRF: chronic renal failure
TGF-β: transforming growth factor-β
MMP: mitochondrial membrane potential
MCP: monocalcium phosphate
Cr: creatinine
UN: urea nitrogen
LTB4: leukotrienes B4
NF-κB: nuclear transcription factor-kappa B
MAPK: mitogen-activated protein kinase
PI3K: phosphatidylinositol 3 kinase
